# Fully Automated Deep Learning System for Bone Age Assessment

**DOI:** 10.1007/s10278-017-9955-8

**Published:** 2017-03-08

**Authors:** Hyunkwang Lee, Shahein Tajmir, Jenny Lee, Maurice Zissen, Bethel Ayele Yeshiwas, Tarik K. Alkasab, Garry Choy, Synho Do

**Affiliations:** Massachusetts General Hospital and Harvard Medical School, Radiology, 25 New Chardon Street, Suite 400B, Boston, MA 02114 USA

**Keywords:** Bone-age, Structured reporting, Artificial neural networks (ANNs), Automated measurement, Automated object detection, Clinical workflow, Computer-aided diagnosis (CAD), Computer vision, Data collection, Decision support, Digital X-ray radiogrammetry, Efficiency, Classification, Machine learning, Artificial intelligence

## Abstract

Skeletal maturity progresses through discrete phases, a fact that is used routinely in pediatrics where bone age assessments (BAAs) are compared to chronological age in the evaluation of endocrine and metabolic disorders. While central to many disease evaluations, little has changed to improve the tedious process since its introduction in 1950. In this study, we propose a fully automated deep learning pipeline to segment a region of interest, standardize and preprocess input radiographs, and perform BAA. Our models use an ImageNet pretrained, fine-tuned convolutional neural network (CNN) to achieve 57.32 and 61.40% accuracies for the female and male cohorts on our held-out test images. Female test radiographs were assigned a BAA within 1 year 90.39% and within 2 years 98.11% of the time. Male test radiographs were assigned 94.18% within 1 year and 99.00% within 2 years. Using the input occlusion method, attention maps were created which reveal what features the trained model uses to perform BAA. These correspond to what human experts look at when manually performing BAA. Finally, the fully automated BAA system was deployed in the clinical environment as a decision supporting system for more accurate and efficient BAAs at much faster interpretation time (<2 s) than the conventional method.

## Introduction

Skeletal maturity progresses through a series of discrete phases, particularly in the wrist and hands. As such, pediatric medicine has used this regular progression of growth to assign a bone age and correlate it with a child’s chronological age. If discrepancies are present, these help direct further diagnostic evaluation of possible endocrine or metabolic disorders. Alternatively, these examinations may be used to optimally time interventions for limb-length discrepancies. While the process of bone age assessment (BAA) is central to the evaluation of many disease states, the actual process of BAA has not changed significantly since the publication of the groundbreaking atlas in 1950 by Greulich and Pyle [1], which was developed from studying children in Ohio from 1931 to 1942.

BAA can be performed either using the Greulich and Pyle (GP) [1] or Tanner-Whitehouse (TW2) [2] methods. The GP method compares the patient’s radiograph with an atlas of representative ages and determines the bone age. The TW2 system is based on a scoring system that examines 20 specific bones. In both cases, BAA requires a considerable time and contains significant interrater variability, leading to clinical challenges when therapy decisions are made based on changes in a patient’s BAA. Attempts have been made to shorten the evaluation process by defining shorthand methods to perform BAA more efficiently; however, these still rely on human interpretation and reference to an atlas [3].

BAA is the ideal target for automated image evaluation as there are few images in a single study (one image of the left hand and wrist) and relatively standardized reported findings (all reports contain chronological and skeletal ages with relatively standardized keywords, like “bone age” or “year old”). This combination is an appealing target for machine learning, as it sidesteps many labor-intensive preprocessing steps such as using Natural Language Processing (NLP) to process radiology reports for relevant findings.

**Fig. 1:**
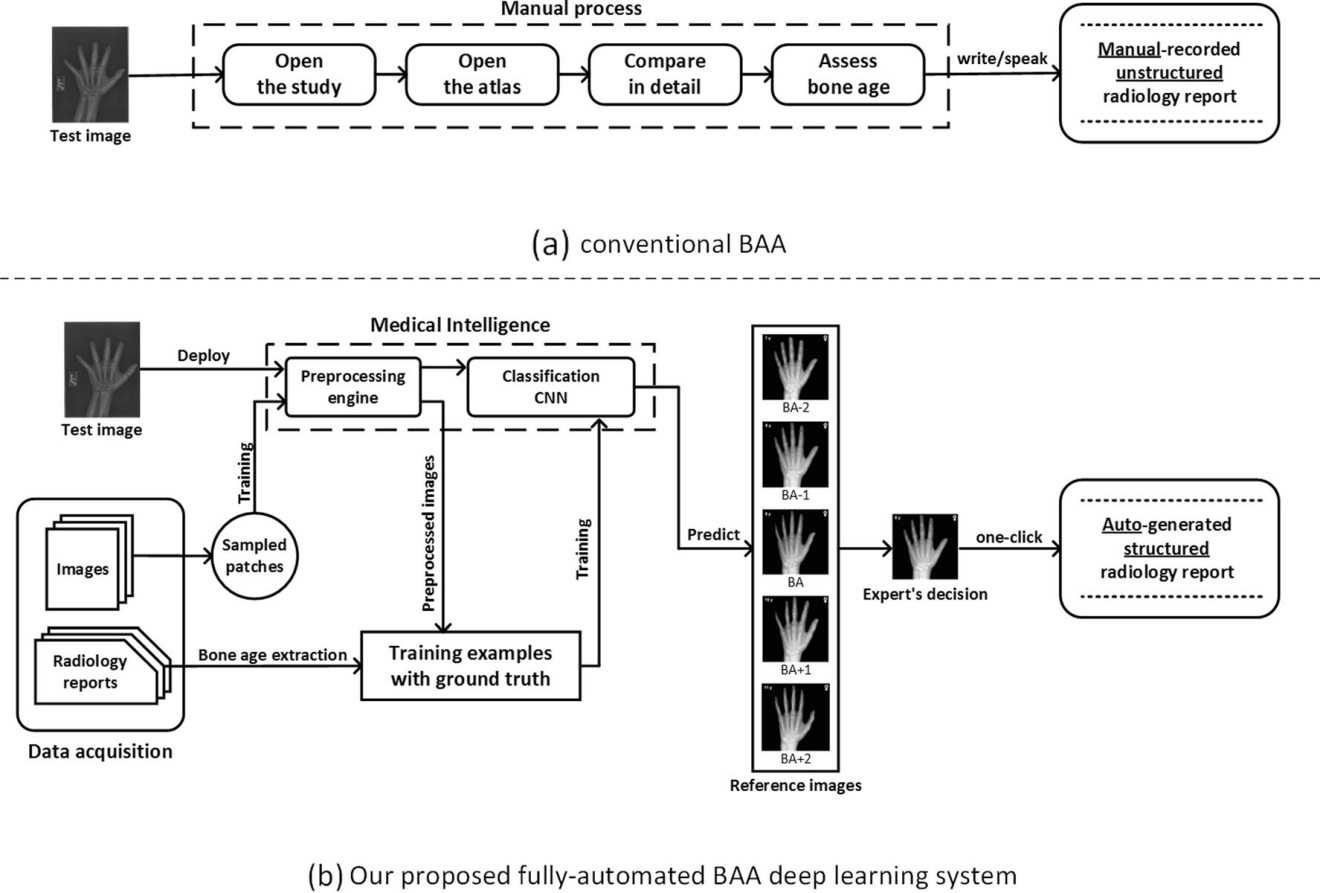
Overview of **a** the conventional GP-BAA methodology and **b** our proposed fully automated BAA deep learning system

Deep learning has proven itself a powerful method for a wide range of computer vision image tasks [4], leading to growing interest in using the technique to replace conventional algorithms using manually crafted features. From using deep CNNs to detect patterns of interstitial lung disease on 2D patches of chest CTs [5] to segmenting the vascular network of the human eye on fundus photos [6], deep CNNs have proven enormously successful since they enable learning highly representative, layered, hierarchical abstractions from image data [7]. In addition to segmentation and detection tasks, many deep learning-based methods are well suited for recognition and classification tasks in medical imaging [8, 9]. However, to the best of our knowledge, a large-scale, fully-automated, data-driven, deep learning approach has not been introduced to reduce human expert interobserver variability and improve workflow efficiency of BAA in any published works. We propose a fully automated deep learning platform to perform BAA using deep CNNs for detection and classification with the deployed system able to automatically generate structured radiology reports as in Fig. 1.

## Method

### Data Preparation

#### Data Collection

IRB approval was obtained for this retrospective study. Using an internal report search engine (Render), all radiographs and radiology reports using the exam code “XRBAGE” were queried from 2005 to 2015. Accession numbers, ages, genders, and radiology reports were collected into a database. Using the open source software OsiriX, DICOM images corresponding to the accession numbers were exported. Our hospital’s radiology reports include the patient’s chronological age and the bone age with reference to the standards of Greulich and Pyle, second edition [1].

### Data Categorization

**Fig. 2:**
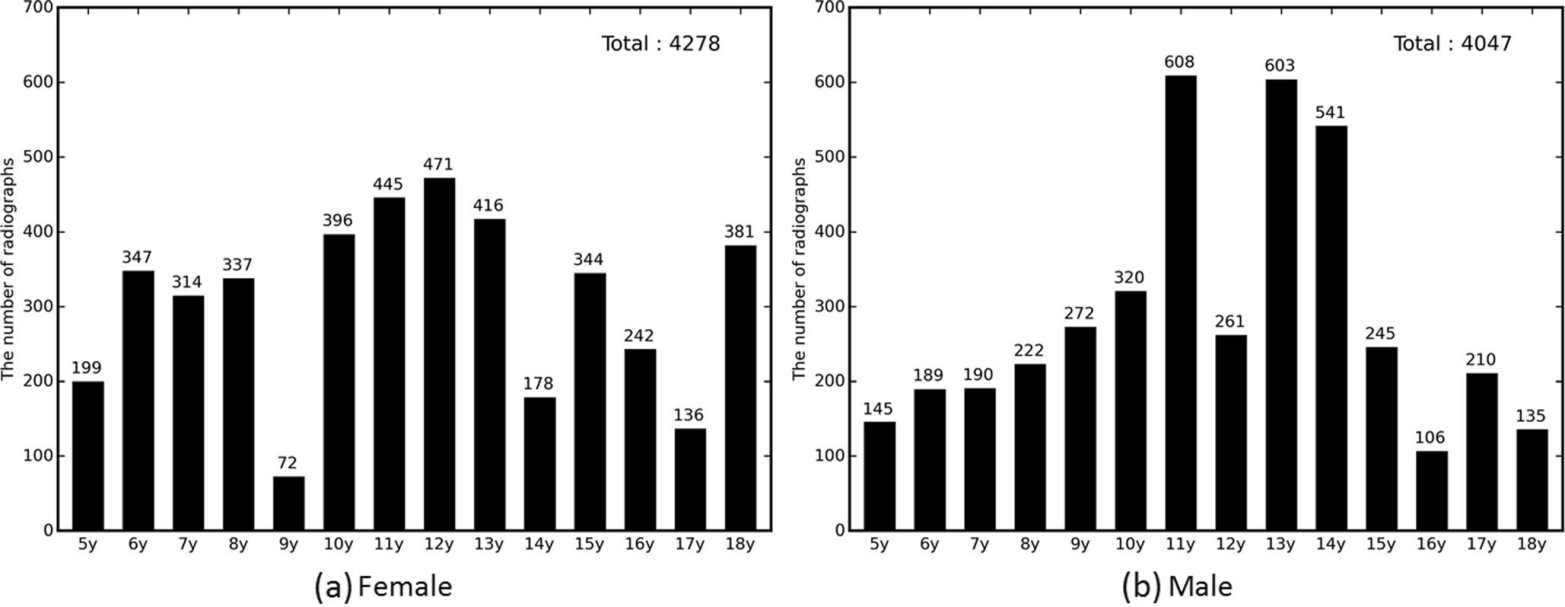
Bone age distribution for radiographs of female and male left hands

Radiographs from patients with chronological age of 5–18 years and skeletally mature (18 years and up) were included in the dataset. In this study, ages 0–4 years were excluded for two reasons. First, there were only a limited amount of radiographs for patients in the 0–4 year-old bracket (298 cases for females and 292 cases for males), which significantly reduced the volume of images usable for training. Second, the overwhelming indication for bone age assessment at our institution is for questions of delayed puberty, short stature, or precocious puberty. These examinations are infrequently performed for patients less than 5 years of age. The reported bone ages were extracted from the radiologist report by determining bone age-related keywords such as “bone age” and “skeletal.” The extracted bone ages were calculated in the form of years, floored, and categorized by year ranging from 5 to 18 years. Skeletally mature cases were considered 18 years [10]. For cases where the reported bone ages were given in a range, we assigned the arithmetic mean of the range as the actual bone age. The total number of studies originally retrieved was 5208 for the female cohort and 5317 for the male cohort. After excluding ages 0–4 years and aberrant cases—right hands, deformed images, and uninterpretable reports—4278 radiographs for females and 4047 radiographs for males were labeled by skeletal age as in Fig. 2.

We randomly selected 15% of the total data for use as a validation dataset and 15% for use as a test dataset. The remainder (70%) was used as training datasets for the female and male cohorts. The validation datasets were utilized to tune hyperparameters to find the best model out of several trained models during each epoch. The best network was evaluated using the test datasets to determine whether the top 1 prediction matched the ground truth, was within 1 year or 2 years. In order to make a fair comparison, we used the same split datasets for each test as new random datasets might prevent fair comparisons.

### Preprocessing Engine

**Fig. 3:**
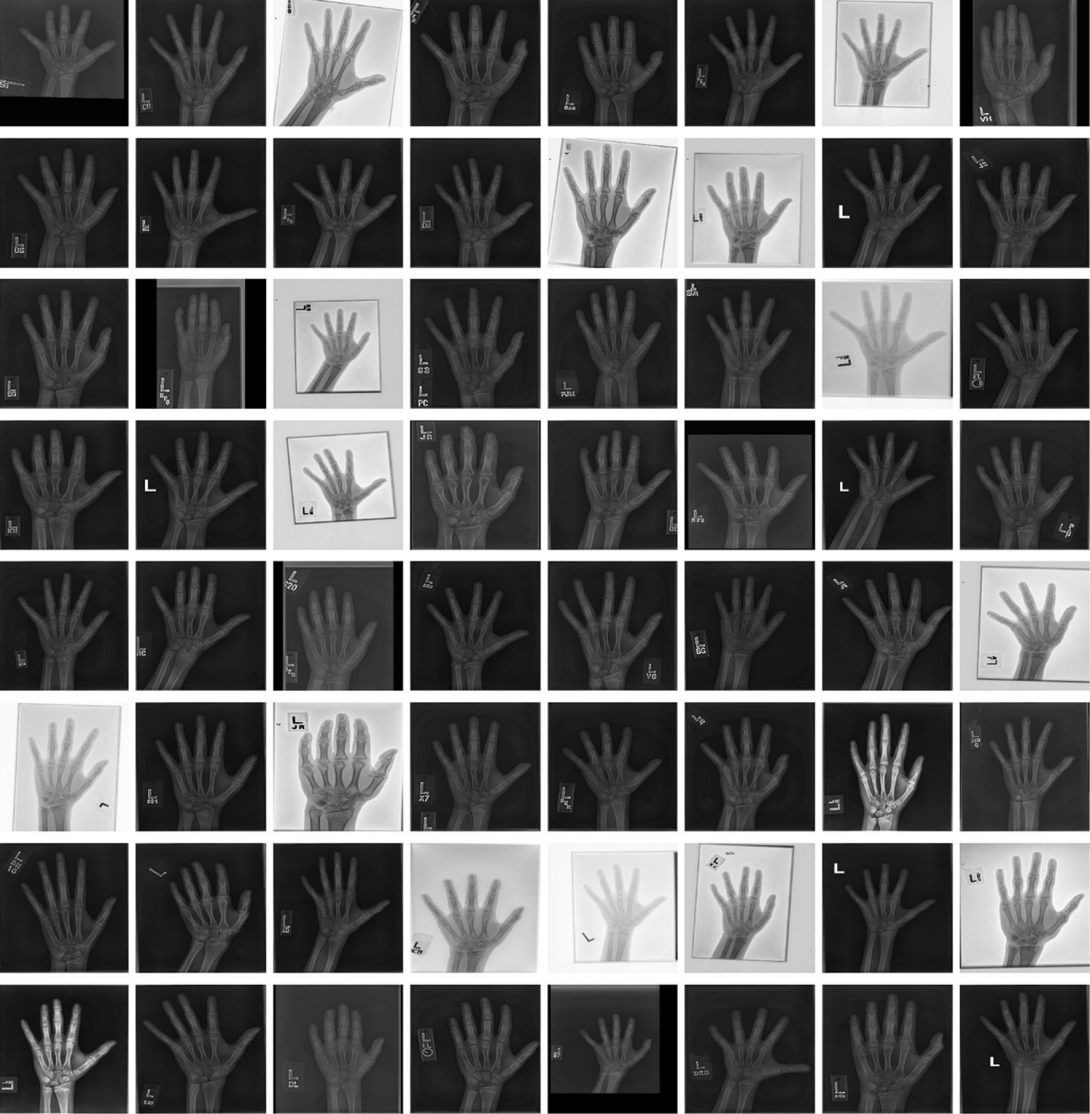
Examples of input radiographs utilized in this work. All images have varying sizes, but they were resized for the purposes of this figure

Input DICOM images vary considerably in intensity, contrast, and grayscale base (white background and black bones or black background and white bones) as shown in Fig. 3. This variance of the training radiographs prevents algorithms from learning salient features. As such, a preprocessing pipeline that standardizes images is essential for the model’s accuracy by eliminating as much unnecessary noise as possible. For this application, bones are the most important features to be preserved and enhanced as they are central to BAAs. Therefore, we propose a novel preprocessing engine that consists of a detection CNN to identify/segment the hand/wrist and create a corresponding mask followed by a vision pipeline to standardize and maximize the invariant features of images.

**Fig. 4:**
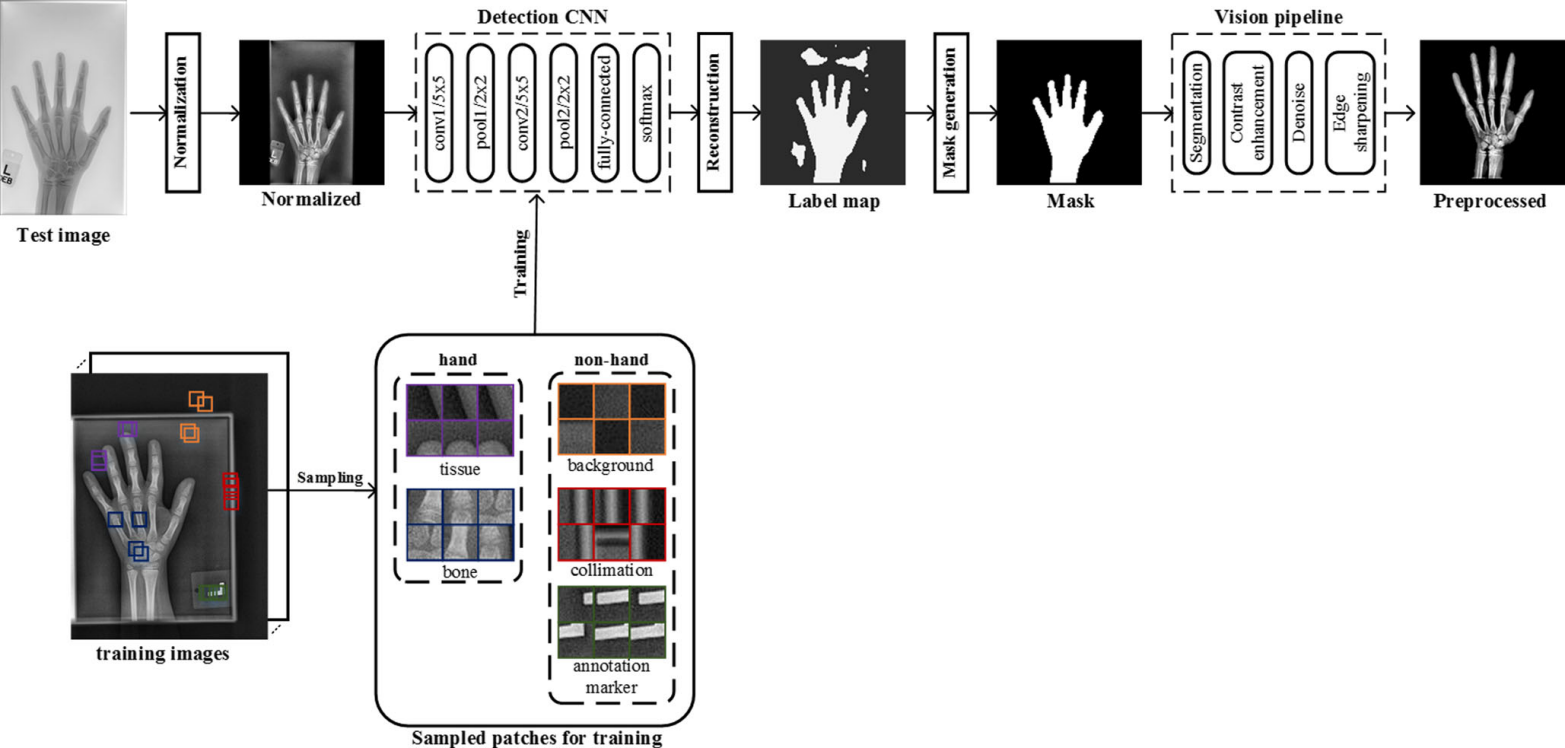
Overview of a deep CNN-based preprocessing engine to automatically detect a hand, generate a hand mask, and feed that into the vision pipeline to standardize images, making the trained automated BAA model invariant to differences in input images

### Normalization

The first step of the preprocessing engine is to normalize radiographs for a grayscale-base and image size before feeding them to the detection CNN. Some images have black bones with white backgrounds and others have white bones with black backgrounds (Fig. 3). Image size varies considerably from a few thousand to a few hundred pixels. To normalize the different grayscale bases, we calculated the pixel-means of 10 × 10 image patches in the four corners of each image and compared them with the half value of the maximum value for a given image resolution (e.g., 128 for 8-bit resolution). This effectively determines whether an image has a white or black background, allowing us to normalize them all to black backgrounds. The next step normalizes sizes of input images. Almost all hand radiographs are height-wise rectangles. Accordingly, we resized the heights of all images to 512 pixels, then through a combination of preserving their aspect ratios and using zero-padding; the widths were all made 512 pixels, ultimately creating standardized 512 × 512 images. We chose this size for two reasons: it needed to be larger than the required input size (224 × 224) for the neural network, and this size is the optimal balance for the performance of the detection CNN and the speed of preprocessing. Larger squares improve the detection CNN performance at the cost of slower deployment time, while smaller squares accelerate the testing time, but they result in worse image preprocessing.

### Detection CNN

There are five different types of objects on hand radiographs: bone, tissue, background, collimation, and annotation markers (Fig. 3). In order to segment the hand and wrist from radiographs, we utilized a CNN to detect bones and tissues, construct a hand/wrist mask, and apply a vision pipeline to standardize images. As shown in Fig. 4, image patches for the five classes were sampled in the normalized images through the use of ROIs. The sampled patches are a balanced dataset with 1 M samples from each class. We used 1000 unique radiographs, which randomly selected from the training dataset, to generate diverse object patches. We used LeNet-5 [11] as the network topology for the detection CNN because the network is an efficient model for coarse-grained recognition of obviously distinctive datasets and used in applications such as MNIST digit recognition [12]. In addition, the network requires small amount of computations and trivial memory space for trainable parameters at deployment time. We trained the model with the set of the sampled patches for 100 epochs using a stochastic gradient descent (SGD) algorithm with 0.01 of the base learning rate decreased as a factor of ten by three steps based on convergence to loss of function. The 25% of training images per class were held out as a validation dataset to select the best model out of epochs.

### Reconstruction

The next step is to construct a label map which contains hand and non-hand regions. For each input radiograph, the detection system slides across the entire image, sampling patches, and records all class scores per pixel using the trained detection CNN. Based on the score records, the highest-score class is labeled to each pixel. After that, a label map is constructed by assigning pixels labeled as bone and tissue classes to a hand label and other pixels to a non-hand label.

### Mask Generation

Most label maps have clearly split regions of hand and non-hand classes, but like an example in Fig. 4, false-positive regions were sometimes assigned to the hand class. As a result, we extracted the largest contiguous contour, filled it, and then created a clean mask for the hand and wrist shown in Fig. 4.

### Vision Pipeline

After creating the mask, the system passes it to the vision pipeline. The first stage uses the mask to remove extraneous artifacts from the image. Next, the segmented region is centered in the new image to eliminate translational variance. Subsequently, histogram equalization for contrast enhancement, denoising, and sharpening filters are applied to enhance the bones. A final preprocessed image is shown in Fig. 4.

### Image Sample Patch Size and Stride Selection

**Fig. 5:**
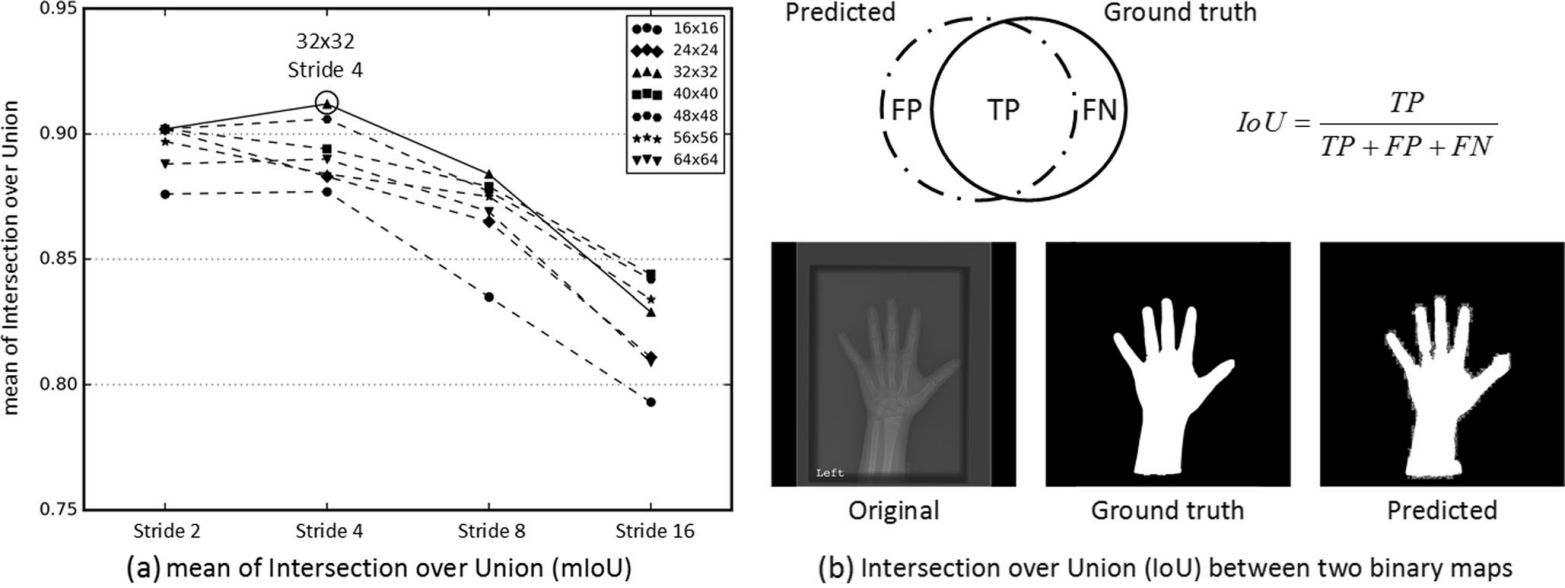
Finding the optimal combination of image patch sizes and strides for optimal mask generation in the preprocessing engine. **a** mean Intersection over (mIoU) results were shown for all combinations of strides (2, 4, 8, 16) and image patch sizes (16 × 16, 24 × 24, 32 × 32, 40 × 40, 48 × 48, 56 × 56, 64 × 64). **b** Representative predicted and ground truth binary maps with the equation for Intersection over Union (IoU) for a single case. mIoU was calculated by taking the arithmetic mean of IoU values for all 280 test cases

Preprocessing performance depends on the size of an image sample patch and the stride by which the detection system moves. We conducted a regressive test to find the optimal image patch size and stride by comparing varying strides (2, 4, 8, 16) and image patch sizes (16 × 16, 24 × 24, 32 × 32, 40 × 40, 48 × 48, 56 × 56, 64 × 64) as shown in Fig. 5a. For this experiment, 280 images representing 10 images per class for females and males were randomly selected from the test dataset to evaluate the preprocessing engine’s performance by calculating the arithmetic mean of Intersection over Union values (mIoU) between the predicted and ground truth binary maps. Based on the results in Fig. 5, a 32 × 32 image patch size and a stride of 4 are the optimal configuration with a mIoU of 0.92.

### Classification CNN

Deep CNNs consist of alternating convolution and pooling layers to learn layered hierarchical and representative abstractions from input images, followed by fully connected classification layers which are then trainable with the feature vectors extracted from the earlier layers. They have achieved considerable success in many computer vision tasks including object classification, detection, and semantic segmentation. Many innovative deep neural networks and novel training methods have demonstrated impressive performance for image classification tasks, most notably in the ImageNet competition [13–15]. The rapid advance in classification of natural images is due to the availability of large-scale and comprehensively annotated datasets such as ImageNet [16]. However, obtaining medical datasets on such scale and with equal quality annotation as ImageNet remains a challenge. Medical data cannot be easily accessed due to patient privacy regulations, and image annotation requires an onerous and time-consuming effort of highly trained human experts. Most classification problems in the medical imaging domain are fine-grained recognition tasks which classify highly similar appearing objects in the same class using local discriminative features. For example, skeletal ages are evaluated by the progression in epiphyseal width relative to the metaphyses at different phalanges, carpal bone appearance, and radial or ulnar epiphyseal fusion, but not by the shape of the hand and wrist. Subcategory recognition tasks are known to be more challenging compared to basic level recognition as less data and fewer discriminative features are available [17]. One approach to fine-grained recognition is transfer learning. It uses well-trained, low-level knowledge from a large-scale dataset and then fine-tunes the weights to make the network specific for a target application. This approach has been applied to datasets that are similar to the large-scale ImageNet such as Oxford flowers [18], Caltech bird species [19], and dog breeds [20]. Although medical images are considerably different from natural images, transfer learning can be a possible solution by using generic filter banks trained on the large dataset and adjusting parameters to render high-level features specific for medical applications. Recent works [21, 22] have demonstrated the effectiveness of transfer learning from general pictures to the medical imaging domain by fine-tuning several (or all) network layers using the new dataset.

### Optimal Network Selection for Transfer Learning

**Fig. 6:**
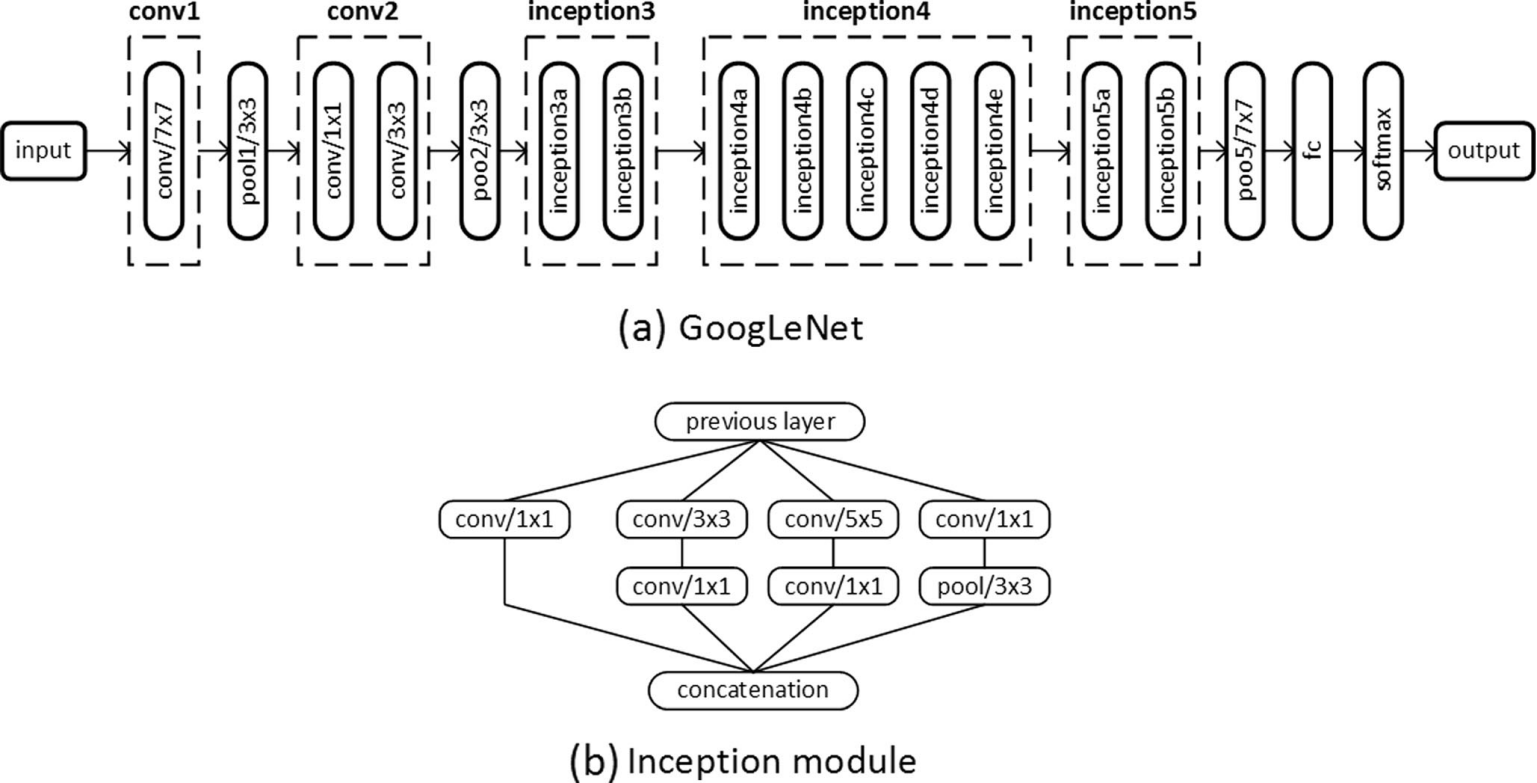
**a** GoogLeNet network topology that we used for this study. **b** The inception module, utilized in GoogLeNet, contains six convolutional layers with different kernel sizes and a pooling layer. All resultant outputs are concatenated into a single output vector

We considered three high-performing CNNs, including AlexNet [13], GoogLeNet [14], and VGG-16 [15], as candidates for our system as they were validated in ImageNet Large Scale Visual Recognition Competition (ILSVRC) [23]. Fortunately, Canziani et al. performed a comparative study between the candidate networks. A summary of their differences is presented in Table 1 [24]. If accuracy is the sole determiner, VGG-16 is the best performer and AlexNet is the worst. However, GoogLeNet utilizes ∼25 times fewer trainable parameters to achieve comparable performance to VGG-16 with a faster inference time. In addition, GoogLeNet is the most efficient neural network [24], particularly because the inception modules described in Figs. 5 and 6, enable the network to have a greater capability to learn hierarchical representative features without many trainable parameters by minimizing the number of fully connected layers.

**Table 1:** Comparisons of the three candidate networks for transfer learning in terms of trainable parameter number, computational requirements for a single inference, and single-crop top 1 accuracy on the ImageNet validation dataset

	No. of trainable parameters	No. of operations needed for a single inference	Single-crop top 1 validation accuracy
GoogleNet [14]	˜̃5M (1×)	˜̃3 G-ops (1×)	˜̃68.00%
AlexNet [13]	˜̃60M (12×)	˜̃2.5 G-ops (0.83×)	˜̃54.50%
VGG-16 [15]	˜̃140M (28×)	˜̃32 G-ops (10.6×)	˜̃70.60%

Numbers from a comparative study conducted by Canziani et al. [24]

### Training Details

We retrieved a pretrained model of GoogLeNet from Caffe Zoo [25] and set about fine-tuning the network to medical images. ImageNet consists of color images, and the first layer filters of GoogLeNet correspondingly comprise three RGB channels. Hand radiographs are grayscale, however, and only need a single channel. As such, we converted the filters into a single channel by taking arithmetic means of the preexisting RGB values. We confirmed that the converted grayscale filters matched the same general patterns of filters, mostly consisting of edge, corner, and blob extractors. After initializing the network with the pretrained model, our networks were further trained using an SGD for 100 epochs with a mini-batch size of 96 using 9 different combinations of hyperparameters, including base learning rates (0.001, 0.005, 0.01) and gamma values (0.1, 0.5, 0.75), in conjunction with a momentum term of 0.9 and a weight decay of 0.005. Learning rate, a hyperparameter that controls the rate of weights and bias change during training a neural network, is decreased by the gamma value by three steps to ensure a stable convergence to loss function. It is challenging to determine the best learning rate because it varies with intrinsic factors of the dataset and neural network topology. To resolve this, we use an extensive grid search for optimal combinations of hyperparameters using the NVIDIA Devbox [26] to find the optimal learning rate schedule.

### Preventing Overfitting (Data Augmentation)

Deep neural networks require a large amount of labeled training data for stable convergence and high classification accuracy. If there is limited training data, deep neural networks will overfit and fail to generalize for target application. This is a particular challenge in medical imaging, as compilation of high quality and well-annotated images is a laborious and expensive process. As a result, several methods are used to decrease the risk of overfitting. Data augmentation is one technique where we synthetically increase the size of the training dataset with geometric transformations, photometric transformations, noise injections, and color jittering [13], while preserving the same image label. Table 2 details the geometric, contrast, and brightness transformations used for real-time data augmentation and the number of possible synthetic images for each. Affine transformations, including rotation, scaling, shearing, and photometric variation were utilized to improve resiliency of the network to geometric variants and variations in contrast or intensity. Rotations ranged from −30 to +30 in 5° increments. Scaling operations were performed by multiplying the width by 0.85–1.0 in 0.01 increments and the height by 0.9–1.0 in 0.01 increments. Shearing was performed by applying an x and y angle ranging from −5 to +5 with an increment of 1°. Brightness was adjusted by multiplying all pixels by a factor ranging from 0.9 to 1.0 with increment of 0.01 and adding an integer ranging from 0 to 10. These transformations were augmented with random switches for each transformation. By using real-time data augmentation, a single image can be transformed into one of 1,107,150,000 images (= 61 * 150 * 121 * 100), preventing image repetition during each epoch. This method does not increase computing time or storage as images for the next iteration are augmented on the CPU while the previous iteration is being trained via the GPU. We excluded random horizontal inversion, frequently utilized for natural images, because BAA only uses left-sided radiographs by convention. We also did not perform random translation as all were centered at the image preprocessing stage.

**Table 2:** Summary of real-time data augmentation methods used in the study

Method	Range	No. of synthetic images
rotate	−30° ≤ rotation angle ≤30°	61
resize	0.85 ≤ width < 1.0, 0.9 ≤ height < 1.0	150
shear	−5° ≤ x angle ≤5°, −5° ≤ y angle ≤5°	121
pixel transform	α* pixel +_β, (0.9 ≤ α ≤ 1.0, 0 < β ≤ 10)	100

Geometric (rotation, resizing, and shearing) and photometric transformations (contrast and brightness) were applied to input images prior to training the network to prevent overfitting

## Results

### Preprocessing Engine

**Fig. 7:**
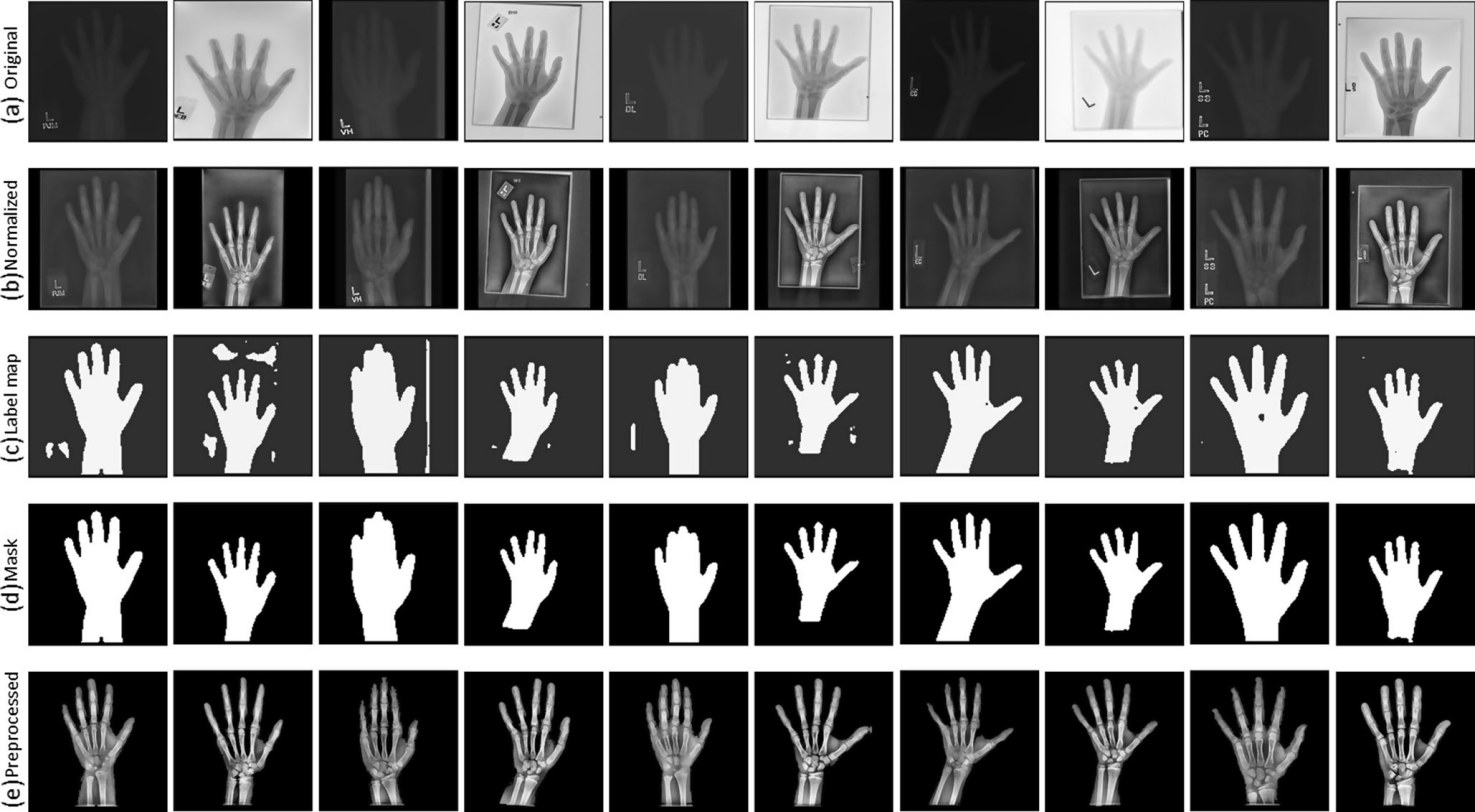
Ten examples at each stage of preprocessing as described in the “Preprocessing engine” section *a* Input radiographs. The images have been transformed to a square shape for consistent layout. *b* Normalized images with consistent grayscale base and image size. *c* Label maps of hand (*white*) and non-hand (*black*) classes. *d* Generated masks for segmentation. *e* Final preprocessed images.

Figure 7 demonstrates the effectiveness of the preprocessing engine at performing image standardization. There is extensive variability among the input images with half the images having white bones on black backgrounds, variable collimation configurations, and presence or absence of side markers. Normalizing the grayscale base and image size produces the images in the second row. The third row presents the constructed label maps used for automatic hand/wrist segmentation used by a second trained CNN. However, the label map cannot be used as a segmentation mask because there are frequently false-positive pixels, such as in the second image of the third row. These pixels can be removed by extracting the largest contour and filling the resulting polygon to create a uniform mask shown in the fourth row. The vision pipeline can then segment the hand and wrist using the generated mask, enhance the bone edges, and denoise the image. The pipeline takes DICOM objects from various vendors with huge differences in appearance then automatically segments, centers, and enhances the images prior to training and deployment.

### Classification CNN

#### Optimal Depth of Fine Tuning

**Fig. 8:**
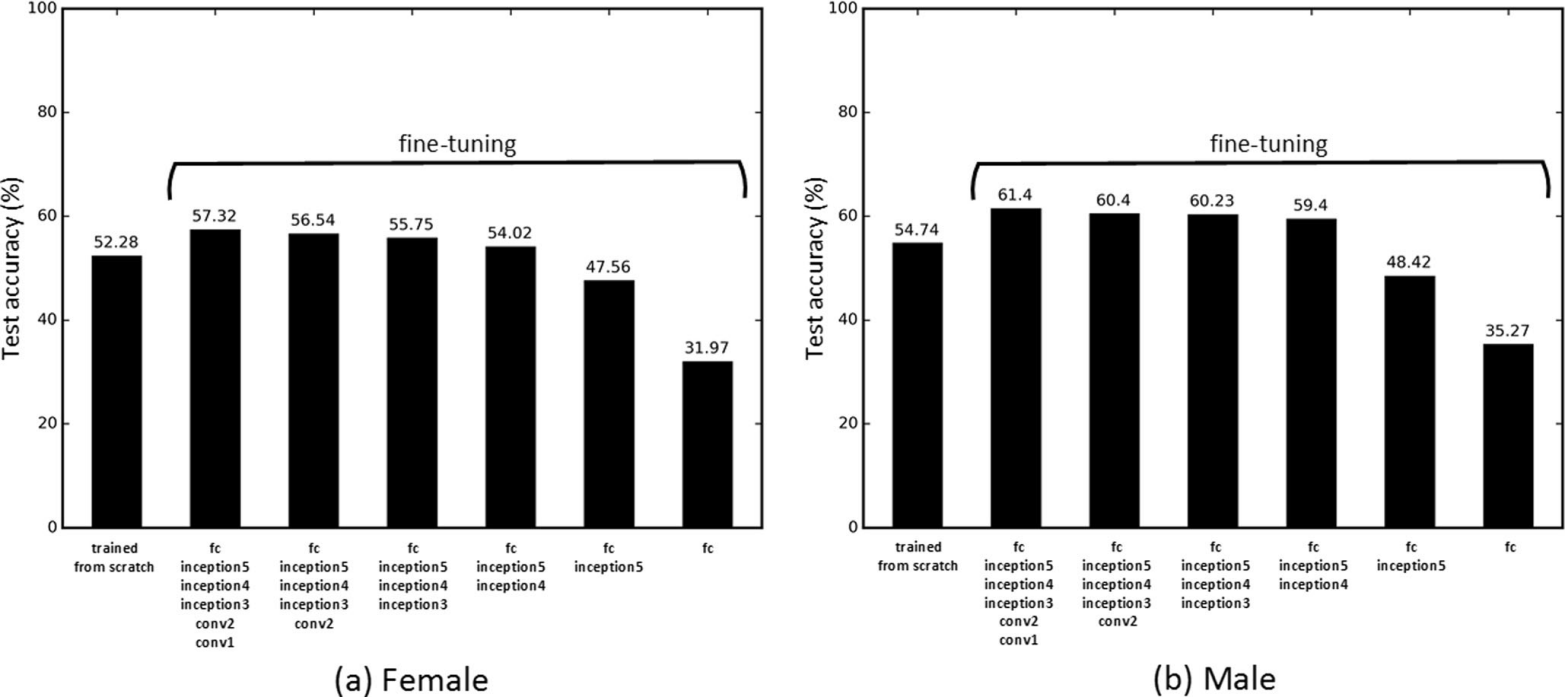
CNN test accuracy with the real-time data augmentation using different styles of training. The “trained from scratch” method trains a CNN with a random weight initialization. Other methods fine-tune the ImageNet pretrained CNNs by incrementally updating weights of each fully connected (fc) layer from inception5 to conv1, detailed in Fig. 6

Tajbakhsh et al. [22] found that a layer-wise fine-tuning schema can find the best performance for a given application with a limited amount of training data in the medical imaging domain. The early layers learn low-level image features, like edges and corners, while the later layers learn higher-level features applicable for the target application [22, 27]. Transfer learning typically requires fine-tuning the later layers to the specific dataset, but it might require fine-tuning early layers, depending on how different the source and target applications are [22]. To find the optimal number of layers requiring adjustment for BAA, we conducted a regressive test by incrementally fine-tuning pretrained CNNs from the last layer to the first. In addition, the CNN was trained from scratch with a random weight initialization to determine whether the fine-tuning method was better than training from scratch. In order to enable a stable convergence of loss function, it is essential to anneal the learning rate over time. Similar to the “Classification CNN” section, a grid search for finding the optimal combination of hyperparameters was conducted to ensure the optimal training parameters. Figure 8 presents test accuracy for the “correct” case, with the real-time data augmentation, for the pretrained CNNs that were fine-tuned for layers ranging from fully connected (fc) to all layers. A base learning rate of 0.005 was determined for the best performing models at fine-tuning tests, and 0.01 was employed for training from scratch. If large learning rates are used for training the pretrained model, well-trained generic features will be overwritten, causing overfitting of the model. We found out that fine-tuning weights of all layers is the best scheme for BAA. Since medical images are markedly different from natural images, all layers must be fine-tuned to generate low-level and high-level features for BAA. When training the network from scratch, there were many cases where the loss function failed to converge, implying that random weight initialization is not a stable training method given the small amount of data.

### Test Accuracy

**Fig. 9:**
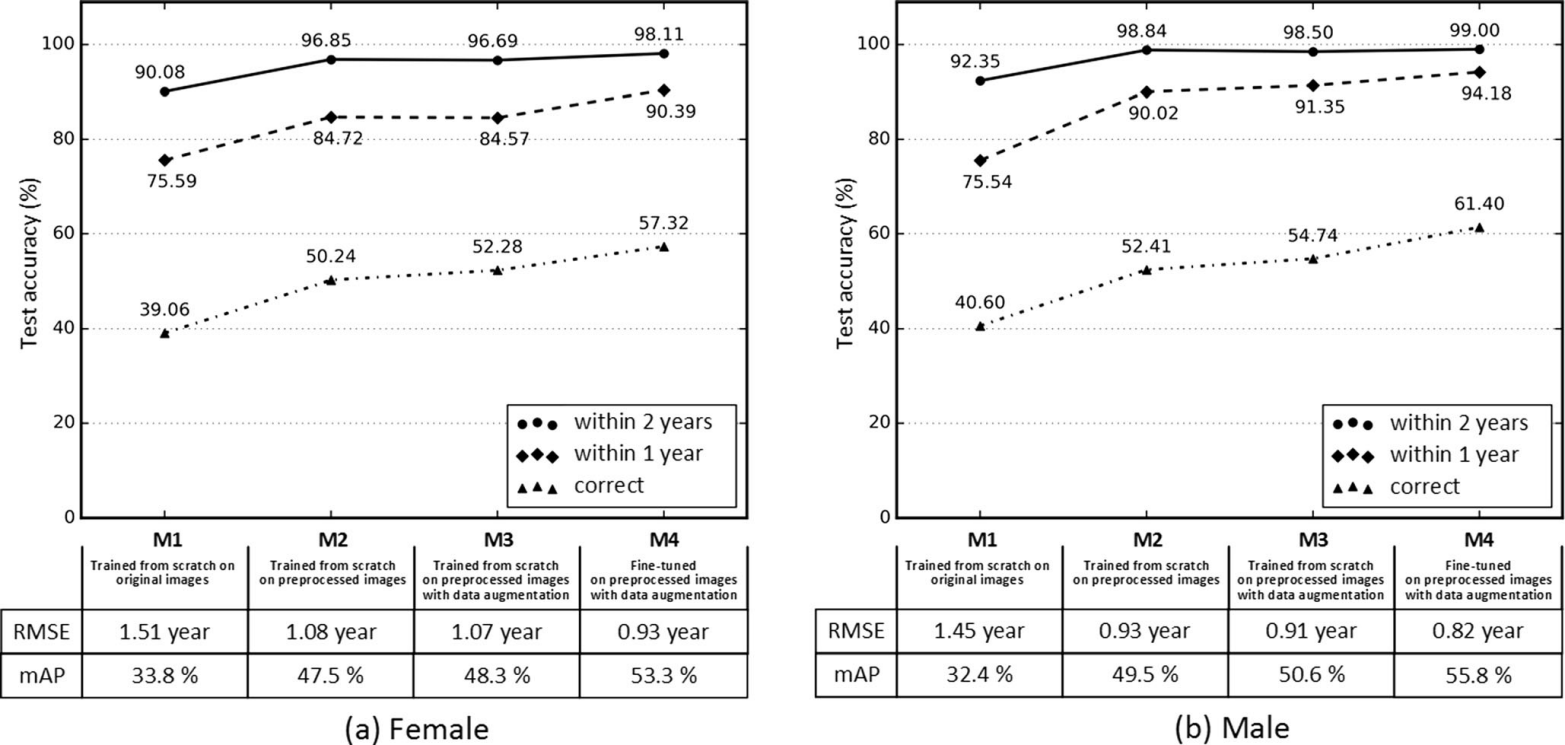
Performance of four different methods (M1–M4) of training for female (**a**) and male (**b**) bone age assessments. M1 trains a CNN from scratch with a random weight initialization on original images down sampled to 224 × 224 pixels. M2 contains images from the automated preprocessing engine. M3 contains synthetically generated images for improving network generalization in addition to M2. M4 fine-tunes an ImageNet pretrained CNN on the preprocessed images with data augmentation turned on. “Correct” corresponds to the case where the prediction of the model is the same as the ground truth. “Within 1 year” and “within 2 years” include the cases where the network’s prediction is within 1 and 2 years, respectively. In addition, root mean squared error (RMSE) and mean average precision (mAP) were reported for the four different models to figure out how robust and well-performing each model is

Test accuracy of the four different methods for female and male BAAs is detailed in Fig. 9. The first model (M1) was the trained CNN with original hand radiographs resized to 224 × 224. Test accuracy was 39.06% for the female cohort and 40.60% for the male cohort. Skeletal ages for the female and male radiographs were assigned an age within 1 year of ground truth 75.59 and 75.54% of the time and within 2 years 90.08 and 92.35% of the time, respectively. The second model (M2) was conducted with preprocessed images, and the third model (M3) was performed by turning on real-time data augmentation while training the network from scratch. Neural network generalization improved with the use of preprocessed and augmented data, with test accuracy increasing by 33.85% for the female cohort and 34.83% for the male cohort. The last model (M4) was the fine-tuned CNN with preprocessed images by turning on real-time augmentation. Test accuracy was 57.32% for the female cohort and 61.40% for the male cohort. BAAs for female radiographs were assigned an age within 1 year of ground truth 90.39% of the time and 98.11% within 2 years. BAAs for male radiographs were assigned an age within 1 year of ground truth (94.18%) of the time and 99.00% of the time within 2 years. Root mean squared error (RMSE) was 0.93 years for females and 0.82 years for males, improved by 62% for the female and 57% for the male cohorts compared to RMSE for M1. Furthermore, mean average precision (mAP) was 53.3% for the female cohort and 55.8% for the male cohort, improved by 57.69% for females and 72.22% for males compared to mAP for M1.

### Visualization

#### Attention Map

**Fig. 10:**
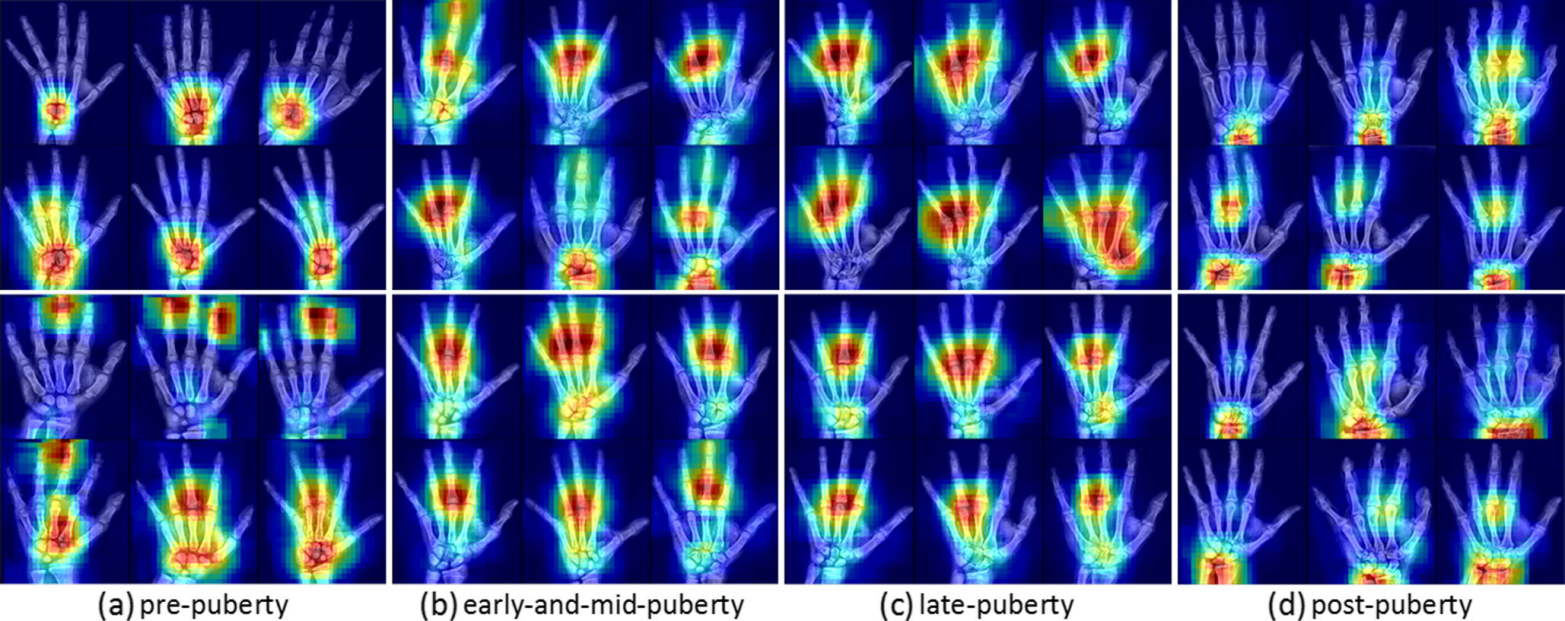
Selected examples of attention maps for female (*upper rows*) and male (*lower rows*) in the four major skeletal maturity stages: prepuberty, early-and-mid puberty, late puberty, and postpuberty stages [10]. Infant and toddler categories were excluded. Six representative attention maps were carefully chosen to represent the general trend for each category. **a** Prepuberty: BAAs from 2 to 7 years for females and 3–9 years for males. **b** Early-and-mid puberty: 7–13 years for females and 9–14 years for males. **c** Late-puberty: 13–15 years for females and 14–16 years for males. **d** Postpuberty: 15 and up for females and 17 years and up for males

Despite their impressive performance at natural image classification, deep neural networks are not well understood. Several approaches for investigating what neural networks use to perform classification have been proposed [27, 28]. We utilized the occlusion method [27] to generate attention maps to find which part of an image is locally significant for fine-grained classification. The occlusion method iteratively slides a small patch across the image, passing occluded input images to the forward network and generating two-dimensional attention maps based on the change in classification probability as a function of occluder position. Only correctly classified input images were selected to determine the important regions of the input images. In Fig. 10, representative attention maps were generated for four major skeletal development stages—prepuberty, early-and-mid puberty, late puberty, and postpuberty [10]—highlighting the important portions of the image which allowed the neural network to perform fine-grained classification. Infant and toddler categories were excluded. Intriguingly, the significant regions for each classification are partially in accordance with the targeted features of each category described in [10]. The prepubertal attention maps (a) focus on carpal bones and mid-distal phalanges. The early-mid and late-pubertal attention maps (b and c) focus less importance on the carpal bones and more on the phalanges, implying that these are more important predictors of BAA than the carpal bones. For postpubertal attention maps (d), importance returns to the wrist, where the radial and ulnar physes are the last to close.

## Discussion

### Comparison with Previous Works

Fully automated BAA has been a goal of computer vision and radiology research for many years. Most prior approaches have included classification or regression using hand-crafted features extracted from regions of interest (ROIs) for specific bones segmented by computer algorithms. Table 3 summarizes four prior attempts at BAA in comparison with our method. Seok et al. [29] utilized a scale invariant feature transform (SIFT) to extract image descriptors and singular value decomposition (SVD) to create fixed-size feature vectors, feeding them into a fully connected neural network. Since they used only a small number of images, their model was not robust to images totally different from their internal dataset. They also did not provide any quantifiable performance metrics. Somkantha et al. [30] selected the carpal bone region using projections in both the horizontal and vertical axes, extracting boundaries of the carpal bones. They extracted five morphological features from the segmented carpal bones, using them for regression with a support vector machine (SVM). This approach is similar to Zhang et al.’s approach [32] in that hand-engineered features were extracted from carpal bones, and the features were used as input for a fuzzy logic classifier. However, this approach is not applicable for children older than 5 to 7 years as the carpal bones are typically fully mature by that age and no longer allow meaningful discrimination beyond that point [10].

**Table 3:** Summary and comparison of prior attempts at automated BAA: dataset, method, salient features, and their limitations

Dataset		Method	Features	Limitations
[29]	24 GP female images	SIFT; SVD Fully connected NN	Fixed-sized features vectors from SIFT description with SVD	Training and validation with limited data; deficiency of robustness to actual images
[30]	180 images from [31]	Canny edge detection Fuzzy classification	Morphological features regarding carpal bones	Not applicable for children above 7 years
[32]	205 images from [31]	Canny edge detection Fuzzy classification	Morphological features regarding carpal bones (Capitate Hamate)	Not applicable for children above 5 years for females and 7 years for males
[33]	1559 images from multiple sources	AAM PCA	Features regarding shapes, intensity, texture of RUS bones	Vulnerable to excessive noise in images chronological age used as input
Our work	8325 images at MGH	Deep CNN transfer learning	Data driven, automatically extracted features	

SIFT scale invariant feature transform, *AAM* active appearance model, *PCA* principle component analysis, *SVD* singular value decomposition, *NN* neural network, *SVM* support vector machine, *RUS* radius ulna short

The most successful attempt to date is BoneXpert [33], a software only medical device approved for use in Europe and the first commercial implementation of automated BAA. BoneXpert utilizes a generative model, the active appearance model (AAM), to automatically segment 15 bones in the hand and wrist and then determine either the GP or TW2 bone age based on shape, intensity, and textural features. Even though BoneXpert reports considerable accuracy for automated BAA, it has several critical limitations. BoneXpert does not identify bone age directly, because the prediction depends on a relationship between chronological and bone ages [29]. The system is brittle and will reject radiographs when there is excessive noise. Prior studies report that BoneXpert rejected around 235 individual bones out of 5161 (4.5%) [34]. Finally, BoneXpert does not utilize the carpal bones, despite their containing discriminative features for young children.

In summary, all prior attempts at automated BAA are based on hand-crafted features, reducing the capability of the algorithms from generalizing to the target application. Our approach exploits transfer learning with a pretrained deep CNN to automatically extract important features from all bones on an ROI that was automatically segmented by a detection CNN. Unfortunately, all prior approaches used varying datasets and provide limited details of their implementations and parameter selection that it is impossible to make a fair comparison with prior conventional approaches.

### How to Improve the System?

#### Classification Accuracy

The trained model in this study achieved impressive classification accuracy within 2 years (>98%) and within 1 year (>90%) for the female and male cohorts. Areas for future improvement abound. We plan to use insights from attention maps and iterative radiologist feedback to direct further learning and improve prediction accuracy. The attention maps reveal key regions similar to what domain experts use to perform conventional BAA; however, it is not certain whether the algorithm uses the exact same features as domain experts. Rather, this method of visualization only reveals that the important regions of the images are similar. The CNN could be using as yet unknown features to perform accurate fine-grained classification which happen to be in the same regions. Further investigation is needed to determine if bone morphology is what the CNN is using for BAA.

However, the algorithm still has room for improvement to provide even more accurate BAA at a faster interpretation time. We down sampled native DICOM images to 8-bit resolution jpegs (224 × 224) to provide a smaller matrix size and use GPU-based parallel computing. In the future, using the native 14-bit or 16-bit resolution images with larger matrix sizes will likely improve the performance of algorithm.

Another approach could be to develop a new neural network architecture optimized for BAA. Recent advanced networks, like GoogLeNet [14], VGGNet [15], and ResNet [35], contain many layers—16 to 152—and run the risk of overfitting given our relatively small amount of training images. Creating a new network topology might be a better approach for BAA which could be more effective than using transfer learning. This would require future systematic study to determine the best algorithm for BAA, beyond the scope of this work.

Lastly, we need to reconsider that bone ages obtained from reports may not necessarily reflect ground truth as BAA is inherently based on subjective analysis of human experts. In some radiology reports, bone ages were recorded as single numbers, a numerical range, or even a time point not in the original GP atlas. In addition, Greulich and Pyle’s original atlas [36] provides standard deviations that range from 8 to 11 months for a given chronological age, reflecting the inherent variation in the study population. As such, not all the ground truths can be assumed as correct. To counter this, the algorithm could be enhanced with an iterative training by applying varying weights to training images based on confidence levels in reports.

### Deployment Time

The proposed deep learning system for BAA will be used in the clinical environment to both more efficiently and more accurately perform BAA. It takes approximately 10 ms to perform a single BAA with a preprocessed image. However, it requires averagely 1.71 s to crop, segment, and preprocess an image prior to classification. Most of the time is consumed by the construction of the label map prior to segmentation. The time could be decreased by exploiting a selective search to process only plausible regions of interest [37]. Additionally, instead of preserving aspect ratios and creating a 512 × 512 pixels image, image warping to a smaller matrix size could reduce the computational time required for segmentation at the cost of eventual output image quality. The optimal balance requires a systematic study, beyond the scope of this work. Although all stages of preprocessing and BAA cannot be performed in real time (<30 ms), net interpretation time (<2 s) is still accelerated compared to conventional BAA, which ranges from 1.4 to 7.9 min [38].

### Clinical Application

Figure 1 details the process of conventional BAA by radiologists and the proposed fully automated BAA system with automated report generation. Radiologists conventionally compare the patient’s radiograph to reference images in the G&P atlas, a repetitive and time-consuming task. Since bone age is evaluated based on a subjective comparison, interrater variability can be considerable. As a result, our system has another major advantage: it reduces interobserver variability for a given examination. Repeated presentations of the same radiograph to the CNN will always result in the same BAA.

Our workflow shows the radiologist a relevant range of images from the G&P atlas with probability estimate of which the algorithm considers the best match. The radiologist then chooses which image he or she thinks is the most accurate BAA, triggering the system to create a standardized report. This system can be seamlessly embedded into the reporting environment, where it provides structured data, improving the quality of health data reported to the EMR.

### Limitations

While our system has much potential to improve workflow, increase quality, and speed interpretation, there are important limitations. Exclusion of 0–4 year olds slightly limits the broad applicability of the system to all ages. Given that 10 years of accessions only included 590 patients of ages 0–4 years (5.6% of the total query), this limitation was felt to be acceptable given the relative rarity of patients in this age range. Eventually, by adding more radiographs to the dataset, we hope to expand our system to include all ages.

Another limitation is our usage of integer-based BAA, rather than providing time-points every 6 months. This is unfortunately inherent to the GP method. The original atlas did not provide consistent time points for assignment of age, rather than during periods of rapid growth, there are additional time points. This also makes training and clinical assessment difficult, given the constant variability in age ranges. This has been a problem that multiple others have tried to correct, such as Gilsanz and Ratib’s work in this area with the Digital Atlas of Skeletal Maturity, which uses idealized images from Caucasian children to provide 29 age groups from 8 months to 18 years of age [10]. While their atlas is more consistent than the GP atlas, it has the serious limitation of not seeing wide clinical adoption, therefore limiting the available training data that we can then use for machine learning.

Because our cohort was underpowered for determinations below annual age determinations, we elected to floor ages in the cases where the age was reported as “X years, 6 months” to maintain a consistent approach to handling all intermediate time points and the fact that chronological ages are naturally counted with flooring. However, this could be introducing error. Retraining the models to account for this by using selectively rounded cases, a higher volume of cases, higher resolution images, or higher powered computer systems to find the optimal combination of settings is beyond the scope of this work but an important future direction.

Lastly, an important consideration is the extent of interobserver variability. Limited directly comparable data is available in the literature regarding interobserver variability in BAA. These estimates range from 0.96 years for British registrars evaluating 50 images using Greulich and Pyle to Tanner’s own publications which suggested manual interpretation using the TW2 system resulted in differences greater than 1 stage ranging from 17 to 33% of the time [38–40]. The most comprehensive open dataset available of hand radiographs with assessment by two raters is the Digital Hand Atlas [31], compiled by the Image Processing and Informatics Lab at the University of Southern California in the late 1990s. All radiographs in that series were rated by two raters, with an overall RMSE of 0.59 years—0.54 years for females, 0.57 years for males, and 0.66 years for all children ranging from 5 to 18 years of age. More recent publication from Korea reported interobserver variation of 0.51 ± 0.44 years by the GP method [41]. These values provide a baseline for the human interobserver variability; however, they may underestimate the true degree of interobserver variability. Our values of 0.93 years for females and 0.82 years for males are comparable to the upper limits of these reported values, keeping in mind that our system does not reject malformed images. While our dataset does provide a rich source from which to perform a rigorous assessment of interobserver variability with multiple raters and experience levels, performing such an analysis is beyond the scope of this work and will be performed as part of future examinations to help guide assessments of system performance.

## Conclusion

We have created a fully automated, deep learning system to automatically detect and segment the hand and wrist, standardize the images using a preprocessing engine, perform automated BAA with a fine-tuned CNN, and generate structured radiology reports with the final decision by a radiologist. This system automatically standardizes all hand radiographs of different formats, vendors, and quality to be used as a training dataset for future model enhancement and achieves excellent average BAA accuracy of 98.56% within 2 years and 92.29% within 1 year for the female and male cohorts. We determined that the trained algorithm assesses similar regions of the hand and wrist for BAA as what a human expert does via attention maps. Lastly, our BAA system can be deployed in the clinical environment by displaying three to five reference images from the G&P atlas with an indication of our automated BAA for radiologists to make the final age determination with one-click, structured report generation.
